# Mitochondrial complex I in focus: mechanisms and therapeutic strategies of urinary system diseases

**DOI:** 10.3389/fphys.2026.1753159

**Published:** 2026-03-10

**Authors:** Fangqiu Yu, Yixuan Li, Ruonan Qu, Zhong Wang, Wenqiang An, Yan Chen, Zhongjin Yue, Wei Wang

**Affiliations:** Department of Urology, Lanzhou University Second Hospital, Lazhou, China

**Keywords:** disease diagnosis, gene mutation, metabolism, mitochondrial complex I, therapies, urinary system diseases

## Abstract

Recent research findings on the role of mitochondrial complex I (CI) in promoting renal cell carcinoma metastasis have been published in *Nature*. Mitochondria, as essential intracellular organelles in mammalian cells, play a pivotal role in orchestrating biological oxidation processes and are crucial for maintaining cellular metabolic homeostasis. Severe mitochondrial dysfunction, particularly involving CI, can lead to the development of urinary system diseases by initiating a cascade of events such as inflammation, impaired mitochondrial autophagy, and related processes. This article explores the involvement of CI in the pathogenesis and progression of urinary system diseases. It begins by introducing fundamental theories related to CI research in urinary system diseases, including its evolution, structure, function, and role in cellular metabolism. The epidemiology of CI-associated urinary system diseases, encompassing both neoplastic and non-neoplastic conditions and their associated risk factors, is subsequently discussed. The article further elaborates on the pathological mechanisms, diagnostic techniques, and therapeutic strategies targeting CI in these diseases. In conclusion, this review addresses the controversies and future directions within this research domain, aiming to provide a comprehensive understanding of the CI in urinary system diseases. It also emphasizes potential avenues for future research and translational applications.

## 1 Introduction

Mitochondria are double-membraned, rod-shaped organelles, ranging from 0.5 to 3 µm in size, and are present in all nucleated aerobic eukaryotic cells (Annesley and Fisher, 2019). They play a crucial role in the bioenergetics of all mammalian cells, with the adenosine triphosphate (ATP) they produce accounting for approximately 90% of the energy required by cells to maintain homeostasis (Brand et al., 2013). In addition to their role in energy production, mitochondria are involved in intracellular signaling pathways and regulate various physiological processes, including apoptosis, calcium homeostasis, and free radical production (Larsson, 2010; Balaban et al., 2005). Mitochondrial dysfunction has been closely linked to cancer and a range of urinary system diseases (Shen Z. et al., 2024). Mitochondrial inheritance is independent and exclusively maternal, as paternal mitochondria are degraded during early embryonic development (Sato and Sato, 2011). The oocyte contains approximately 100 times more mitochondria than typical somatic cells, with each mitochondrion harboring about two to ten copies of its mitochondrial genome (mtDNA) (Babayev and Seli, 2015). Recent research highlights the critical involvement of mitochondria in various diseases. Notably, an increased mtDNA copy number relative to nuclear DNA has been strongly associated with an elevated risk of cancer and is linked to several urological diseases (Bezwada et al., 2024).

The mitochondrial electron transport chain is essential for the conversion of metabolic energy into ATP (Fiedorczuk and Sazanov, 2018). During catabolic processes, electrons are captured as NADH and introduced into the chain via the proton-pumping enzyme NADH: ubiquinone oxidoreductase (complex I, CI), the largest and most complex component of the system. These electrons are then transferred to the proton pump ubiquinol–cytochrome c oxidoreductase (complex III, functioning as the dimer CIII) through ubiquinone, a lipid-soluble electron carrier, and ultimately to cytochrome c oxidase (complex IV, CIV) via cytochrome c, where molecular oxygen is reduced to water (Moser et al., 2006). The orchestrated activity of these complexes facilitates the translocation of protons from the mitochondrial matrix to the intermembrane space, thereby establishing an electrochemical gradient that drives ATP synthase (complex V) to synthesize ATP (Walker, 2013). It is estimated that approximately 30% of mitochondrial energy metabolism diseases are attributable to mutations in nuclear or mitochondrial genes encoding subunits of CI (Kirby et al., 1999). On a molecular level, nearly all pathological conditions stem from impairments in oxidative phosphorylation (OXPHOS); however, the clinical manifestations exhibit considerable variability in type and severity among patients (Garcia-Heredia and Carnero, 2015). CI has been implicated in a range of biological processes, including carcinogenesis, aging, and the pathogenesis of neurodegenerative diseases (Urra et al., 2017).

This review emphasizes the molecular mechanisms underlying CI-related urological diseases and explores the diagnostic and therapeutic potential of mutation identification in these contexts. For the first time, the current understanding of oxidative stress signaling, immune-metabolic interaction, and CI-mediated metabolic reprogramming in urinary system diseases is compiled in this review. It presents CI as a target for medication as well as a disease amplifier. Fill the gap for a systematic review focusing on CI in urinary system diseases.

## 2 Mitochondrial CI in urinary system diseases: basic theories

### 2.1 Mitochondrial CI structure, assembly, and core function

Structurally, CI exhibits an L-shaped configuration consisting of a hydrophilic peripheral arm and a membrane-embedded arm. In humans, CI contains 44 subunits, including 14 core components responsible for NADH oxidation, ubiquinone reduction, and proton translocation, as well as about 30 accessory subunits that provide structural stability but are not directly involved in energy conversion (Fiedorczuk et al., 2016; Letts and Sazanov, 2015; Hirst, 2013). The primary function of CI is to generate reactive oxygen species (ROS): Superoxide (O_2_^−^) is produced by electron leakage at the flavin mononucleotide and Q-sites, especially during reverse electron transfer from succinate-energized ubiquinone (Kussmaul and Hirst, 2006). Excessive CI-derived ROS produce oxidative damage, a key mechanism in ischemia-reperfusion injury, neurodegeneration, and diabetes consequences, whereas low-level ROS fulfill signaling activities (Schieber and Chandel, 2014). Converting NADH to NAD^+^, which can contribute to both physiological and pathological alterations in the body from a metabolic standpoint, is another role of mitochondrial CI.

Beyond its core catalytic subunits, mitochondrial CI function critically depends on a network of biochemical partners that regulate its assembly, stability, and activity. The biochemical dependencies of CI create a network of functional interactions that extend far beyond the 44 core subunits. Cardiolipin provides the structural membrane environment; scaffolding proteins organize CI into functional supercomplexes; assembly factors ensure proper biogenesis; and post-translational modifications enable dynamic regulation (Janssen et al., 2006). Disruption of any node in this network can impair CI function, creating disease phenotypes that may be indistinguishable from primary CI subunit mutations.

#### 2.1.1 Cardiolipin: the mitochondrial signature phospholipid

Cardiolipin (CL) is a unique diphosphatidylglycerol phospholipid found almost exclusively in the inner mitochondrial membrane, where it constitutes 15%–20% of total phospholipids. Its unique structure—four acyl chains and two negative charges—allows it to play crucial roles in supercomplex organization and CI function (Snoke et al., 2022). Phospholipid remodeling enzymes dynamically control the acyl chain composition of CL. Linoleic acid must be incorporated into CL by the phospholipid acyltransferase tafazzin (TAZ) in order to produce the mature L4CL species. Because PTECs in the kidney have large quantities of TAZ and mature L4CL, CL remodeling is especially active in these cells (Reynolds et al., 2023). Rapid CL oxidation and TAZ inactivation brought on by ischemic AKI result in CI instability prior to discernible cell death. In preclinical studies, pharmacological preservation of CL integrity—using mitochondria-targeted antioxidants such as SS-31 (elamipretide) or TAZ activators—protects CI function and reduces renal damage (Garlid et al., 2020).

#### 2.1.2 Scaffolding proteins: organizing CI in respiratory supercomplexes

Instead of operating independently, CI is arranged into higher-order assemblies known as respiratory supercomplexes, or “respirasomes.” These interactions are mediated by scaffolding proteins, which produce functional units that maximize electron transport and reduce the generation of ROS. HIGD1A activation preserves residual CI activity in hypoxic PTECs, averting catastrophic ATP depletion. Ischemic kidney damage is made worse by genetic deletion of HIGD1A, but protection is provided by overexpression (Zhu et al., 2023). A scaffolding protein called COX7A2L (also called SCAF1) specifically encourages CIII2-CIV contact, which indirectly influences CI by improving the downstream electron acceptor environment. Even though CI is physically intact, recent research shows that COX7A2L deletion modifies CI kinetics via altering the local ubiquinone/ubiquinol ratio (Hanna et al., 2025).

#### 2.1.3 Scaffolding proteins: organizing CI in respiratory supercomplexes

At least 14 specialized assembly factors are involved in the highly coordinated process of CI assembly, which involves chaperoning subunits through intermediate complexes. Isolated CI insufficiency is caused by mutations in these components, which frequently result in tissue-specific symptoms that highlight cell type-dependent assembly needs. NDUFAF1, NDUFAF2, and NDUFAF are early assembly factors. The N-module (NADH-binding) and Q-module (quinone-binding) subunits create a Q-module intermediate at the start of the assembly process (Laube et al., 2024). By stabilizing this intermediate, NDUFAF1 stops unstable subunits from degrading too quickly. Membrane arm subunit integration is then encouraged by NDUFAF2 and NDUFAF3, with NDUFAF3 being especially necessary for MT-ND1 insertion (Cabrera-Orefice et al., 2021).

#### 2.1.4 Post-translational modifications: dynamic CI regulation

CI activity is dynamically regulated by reversible post-translational modifications that respond to metabolic state and cellular stress. On the one hand, by neutralizing the positive charge necessary for proton translocation, lysine acetylation of the Cl subunits, especially NDUFS1 and NDUFS3, prevents complex function (Protasoni and Zeviani, 2021). When these acetyl groups are removed by the mitochondrial sirtuin SIRT3, CI is activated in response to food shortage. Reduced CI activity, elevated oxidative stress, and accelerated aging-related traits are all seen in SIRT3 deletion animals (Ilari et al., 2020). By maintaining CI function in the kidney during hyperglycemic stress, SIRT3 guards against diabetic nephropathy (Młynarska et al., 2024). On the other hand, Cl subunits are phosphorylated by many kinases, forming regulatory nodes that combine Cl function with cellular signaling. In response to hormonal stimulation, NDUFS4 is phosphorylated by cAMP-dependent protein kinase PKA, which increases CI activity (Papa et al., 2002). Multiple Cl subunits are phosphorylated by the energy-sensitive kinase AMPK, which both persistently promotes mitochondrial biogenesis and acutely inhibits activity during energy stress to save ATP (Yuan et al., 2020).

### 2.2 Mitochondrial CI and its role in cellular metabolism of the urinary system

Mitochondrial CI is fundamental for cellular energy metabolism within the urinary system. The genitourinary system’s various cell types use mitochondrial CI for diverse metabolic purposes, which reflect their unique physiological roles and energy requirements. Because of their evolutionary adaption to particular microenvironments and functional needs, the kidney, urinary tract, and prostate each have diverse cell populations with distinct CI dependencies. Designing targeted therapeutic approaches and understanding disease pathogenesis depend on an understanding of these cell-specific metabolic characteristics.

#### 2.2.1 Renal tubular epithelial cells: extreme CI dependence and metabolic vulnerability

Among all nephron segments, the proximal tubule epithelial cells (PTECs) stand out as the most CI-dependent cell type, a feature intrinsically linked to their massive reabsorptive workload and high mitochondrial density (Bhargava and Schnellmann, 2017). About 70% of filtered water, 80% of salt, and almost all of the glucose, amino acids, and bicarbonate from the glomerular filtrate are reabsorbed by PTECs, which line the initial part of the nephron (Weinberg et al., 2000). According to estimations, PTECs use two to three times as much ATP per cell as hepatocytes or fibroblasts due to their exceptional transport capability. These cells are completely dependent on continuous OXPHOS driven by CI because the Na+/K+-ATPase alone is responsible for 60%–70% of basal ATP consumption. This energy requirement is reflected in the mitochondrial architecture of PTECs. Each PTEC has 2,000–3,000 mitochondria that are closely spaced in the basal cytoplasm to create a “power grid” next to the basolateral membrane, which is home to Na+/K + -ATPase (Nigam, 2015). This spatial arrangement ensures effective energy coupling by reducing ATP diffusion lengths.

#### 2.2.2 Glomerular cells: specialized CI functions in filtration barrier maintenance

Podocytes, mesangial cells, and glomerular endothelial cells are the three specialized cell types found in the glomerulus. Each of these cell types has specific CI requirements that correspond to their various structural and functional responsibilities.

Microfilaments (actin), intermediate filaments (synaptopodin, vimentin), and microtubules are the three interrelated systems that make up the podocyte cytoskeleton. Actin dynamics at the foot processes are very energy-intensive, needing ATP for chaperone-mediated protein folding, myosin-based contractility, and polymerization/depolymerization. Maintaining the correct folding of cytoskeletal and signaling proteins in podocytes requires the production of the mitochondrial chaperonins HSP60 and HSP10, which are dependent on ATP produced from CI (Gbadegesin et al., 2014).

Mesangial cells provide structural support and phagocytic clearing of trapped debris in the glomerulus’ intercapillary region. Depending on their activation state, these cells show varying CI dependency (Qi et al., 2019). While proliferative triggers, such as hyperglycemia in diabetes or immune complex deposition in glomerulonephritis, significantly increase CI activity to promote matrix formation and cell division, quiescent mesangial cells rely somewhat on OXPHOS.

The fenestrated barrier that permits free filtration of water and tiny solutes while keeping proteins is maintained by glomerular endothelial cells. Through endothelial nitric oxide synthase, which needs NADPH generated by the pentose phosphate pathway powered by CI-derived ATP, CI function in these cells facilitates nitric oxide generation. Endothelial dysfunction, thrombosis, and glomerular ischemia are all encouraged by CI failure, which lowers NO bioavailability (Apodaca et al., 2012). The thrombotic microangiopathies observed in severe forms of mitochondrial illness are partly caused by this mechanism.

#### 2.2.3 Urothelial cells: metabolic plasticity and the CI switch

The bladder urothelium exists in two functionally distinct states with correspondingly different CI activity. The urothelium is swollen and comparatively hypoxic during urine storage, and the surface layer’s umbrella cells adopt a glycolytic metabolism with reduced CI activity. This metabolic arrangement preserves oxygen, reduces the generation of ROS in a low-oxygen environment, and keeps ATP levels high enough to maintain the barrier. A proliferative reserve for urothelial renewal is provided by the basal and intermediate cell layers, which maintain a moderate level of CI activity (Khandelwal et al., 2008). Mechanical stretch causes a significant metabolic change when the bladder contracts and voids. PI3K-Akt-mTORC1 signaling initiates within minutes, promoting mitochondrial biogenesis, CI assembly, and PGC-1α upregulation. The energy surge needed for membrane trafficking, tight junction remodeling, and cell volume modulation is produced when ATP generation switches from glycolysis to OXPHOS. This “CI switch” is completely reversible; metabolism reverts to the storage mode when the bladder is refilled (Vats et al., 2006).

#### 2.2.4 Prostate epithelial cells: zonal metabolic specialization

Extremely high CI activity is found in normal prostate epithelial cells, especially in the peripheral and central zones. This activity is predominantly used to support citrate synthesis and secretion rather than ATP production. The specific role of prostatic fluid, which has a 1000-fold higher concentration of citrate than serum, is reflected in this “aerobic glycolysis-like” phenotype (Costello and Franklin, 2005).

Coordinated cytosolic and mitochondrial enzymes are involved in the metabolic pathway. In mitochondria, full oxidation of glucose and fatty acids produces ATP through CI-driven proton pumping. Citrate is simultaneously exported to the cytosol via the mitochondrial citrate carrier (SLC25A1), where ATP citrate lyase breaks it down into acetyl-CoA and oxaloacetate (Mills et al., 2016). While oxaloacetate is transformed back into pyruvate to produce NADPH, acetyl-CoA facilitates membrane lipid synthesis for secretory vesicles. Sperm motility and semen liquefaction depend on this shortened TCA cycle, which uses citrate export instead of full oxidation. However, it is energetically costly, requiring 18 ATP per citrate molecule. This metabolic program is coordinated by the androgen receptor, which directly activates the transcription of genes encoding ATP citrate lyase, the citrate carrier, and CI subunits (Damodaran et al., 2019). Prostate atrophy results from the collapse of this secretory metabolism caused by androgen deprivation, whether it be by surgery or medication.

### 2.3 Common causes and general consequences of CI dysfunction

The proton-translocating CI of the mitochondrial respiratory chain is one of the largest and most complex membrane-bound protein assemblies (Zickermann et al., 2015). It plays a crucial role in oxidative energy production within eukaryotic cells, and impairments in its function are associated with various hereditary and degenerative diseases. High-resolution X-ray crystallography has elucidated the architecture of CI, identifying the central subunits responsible for its bioenergetic activity (Baradaran et al., 2013; Hunte et al., 2010; Efremov et al., 2010). Additionally, mitochondrial-encoded components are integral to CI function. A recently discovered translational isoform of Fas-activated serine/threonine kinase (FASTK) localizes with mitochondrial RNA granules and is vital for the synthesis of ND6 mRNA, a subunit of the NADH dehydrogenase complex encoded by mitochondrial genes (Gonçalves, 2019). The deletion of FASTK in cultured cells or *in vivo* models results in a selective loss of ND6 transcripts and a subsequent decrease in CI activity (Jourdain et al., 2015). Importantly, the redox centers and the proton translocation machinery are spatially separated, with the ubiquinone reduction site located deep within the hydrophilic domain (Wirth et al., 2016). Structural investigations of CI from Yarrowia lipolytica have yielded evidence corroborating the previously proposed two-state stabilization mechanism, wherein ubiquinone redox reactions trigger conformational changes that facilitate proton pumping (Radermacher et al., 2006).

Over recent decades, research focusing on mitochondrial CI in the context of urinary system diseases has made significant progress. Initial studies primarily sought to elucidate the fundamental roles of CI in cellular energy metabolism. Neurons, with their exceptionally high energy demands, are particularly reliant on the mitochondrial OXPHOS system (Giachin et al., 2016). As the largest enzyme within the electron transport chain (ETC), CI is not only pivotal for ATP production but also serves as a major source of ROS (Jeong and Seol, 2008). Recent advancements in the resolution of the three-dimensional structure of CI have provided new insights into its assembly process. Several chaperone proteins have been identified as essential for the maturation and stability of holo-CI, despite their absence from the final complex (Wirth et al., 2016). CI dysfunction is the most common form of OXPHOS defect in humans and is frequently linked to anomalies in its assembly (Torraco et al., 2015). Despite significant advancements, the intricate mechanisms underlying CI biogenesis remain inadequately elucidated, thereby constraining our understanding of how impairments in the ETC undermine cellular integrity within the urinary system.

Research on mitochondrial CI in the context of urinary system diseases has broadened to encompass its involvement in various pathological conditions. Notably, alterations in mitochondrial CI have been associated with common neurodegenerative diseases prevalent among adults and the elderly (Fato et al., 2008). Although the primary focus of research has been on neurodegenerative diseases, the insights gained offer valuable implications for understanding CI’s role in urinary system diseases, given the reliance of both systems on optimal mitochondrial function. Furthermore, evidence from multiple laboratories indicates a negative correlation between ROS generated by CI and lifespan, underscoring CI’s pivotal role in regulating longevity (Stefanatos and Sanz, 2011). These findings imply that, in the context of urinary system diseases, ROS production mediated by mitochondrial CI may contribute to disease pathogenesis and affect the functional integrity of the urinary system.

## 3 Mitochondrial CI and non-neoplastic of urinary system diseases

**FIGURE 1:**
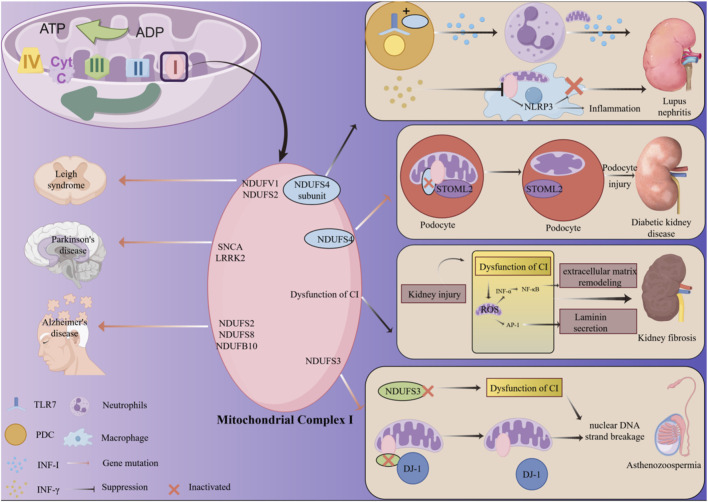
The mechanism of action of mitochondrial CI in urinary system benign diseases. In lupus nephritis, Toll-like receptor 7 (TLR7) binds to the NDUFS4 subunit of the mitochondrial CI, thereby enhancing IFN-I production. Neutrophils can secrete IFN-I via the mitochondrial pathway. IFN-γ disrupts CI activity within macrophages, leading to activation of the NLRP3 inflammasome, which exacerbates inflammatory responses and apoptosis, thereby worsening lupus nephritis. In diabetic kidney disease (DKD), mutations in the NDUFS4 gene within the CI cause STOML2 to dissociate from it, leading to impaired mitochondrial cristae formation in podocytes and thereby contributing to the development of DKD. In kidney fibrosis, CI dysfunction leads to the accumulation of reactive oxygen species, which activates NF-κB and AP-1. NF-κB promotes laminin secretion, while AP-1 activates TGF-β1 to induce extracellular matrix remodeling. This ultimately results in the formation of fibrosis within the kidney. In asthenospermia, reduced expression of the NDUFS3 subunit in CI impairs DJ-1’s ability to protect mitochondria, leading to nuclear DNA fragmentation and ultimately diminished sperm motility.

Mitochondrial CI, the largest enzyme within the OXPHOS system, is composed of 44 distinct subunits encoded by both nuclear and mitochondrial genes. A multitude of pathogenic mutations have been documented within these genes (Fassone and Rahman, 2012; Rodenburg, 2011). In various mitochondrial diseases, defects in genes encoding either the structural subunits or the assembly factors of CI are identified as primary contributors to CI dysfunction (Nouws et al., 2012). Such mutations typically lead to reduced enzymatic activity, which in turn induces cellular-level disturbances, including altered mitochondrial morphology, disrupted membrane potential, and increased production of ROS (Rodenburg, 2016). Mutations in CI genes are frequently associated with degenerative diseases. Leigh syndrome (LS), a subacute necrotizing encephalomyelopathy, exemplifies a progressive neurodegenerative disorder. In a study by Samantha et al., radiological, biochemical, and molecular data from six LS patients were analyzed through gene sequencing, revealing novel mutations in NDUFV1 and NDUFS2, which encode CI subunits (Marin et al., 2013). Subsequent identification of mutations in mtDNA genes MTND2, MTND3, and MTND5 was also reported (Bakare et al., 2021) (Figure 1). Parkinson’s disease (PD) and Alzheimer’s disease (AD) are the most prevalent neurodegenerative diseases. PD is characterized by a gradual decline in motor and non-motor functions, while AD is marked by progressive memory impairment that ultimately leads to cognitive decline severe enough to interfere with daily living activities (Aggarwal and Mielke, 2023; Subrahmanian and LaVoie, 2021; Cenini and Voos, 2019). Both diseases have been linked to mtDNA mutations: PD is associated with alterations in the SNCA and LRRK2 genes, and AD with variants in the NDUFS2, NDUFS8, and NDUFB10 genes (Dar et al., 2023) (Figure 1). Type 2 diabetes (T2D) results from a multifactorial metabolic syndrome, with its complications posing more significant challenges than the disease itself. In terms of treatment and healthcare, T2D represents a substantial global burden, underscoring the necessity of identifying innovative research avenues to better manage T2D and its complications (Whiting et al., 2011; Liu et al., 2010). Case studies utilizing mtDNA sequencing and genetic analyses have identified mutations in the ATPase 8, ND1, and ND5 genes, which are associated with diabetic peripheral neuropathy (Elango et al., 2014; Li et al., 2008). These findings underscore the role of mtDNA in non-neoplastic diseases.

Furthermore, dysfunction of CI has been implicated in non-neoplastic diseases of the urinary system. For example, studies involving the deletion of the essential CI subunit NDUFS4 have demonstrated that mitochondrial disturbances can inhibit macrophage activation and inflammation while simultaneously enhancing osteoclast differentiation and bone resorption through both cell-autonomous and systemic pathways (Jin et al., 2014). These observations prompt the investigation of whether similar mechanisms may be present in non-neoplastic urinary system diseases, where mutations in CI genes could potentially initiate or exacerbate disease onset.

### 3.1 Mitochondrial CI and lupus nephritis

Systemic lupus erythematosus (SLE) is an autoimmune disorder characterized by its impact on multiple organ systems. A defining feature of SLE is the excessive production of autoantibodies, leading to the formation and deposition of immune complexes, which subsequently cause tissue and organ damage, particularly affecting the kidneys and cardiovascular system (Wolf and Ryan, 2019; Bartels et al., 2014). Therefore, the issue of lupus-related complications in clinical practice is highly challenging, and there is an urgent need for corresponding treatment approaches. Mitochondria, as central regulators of immune cell metabolism, play a significant role in various diseases. Recent studies have identified mitochondrial dysfunction as a key contributor to the pathogenesis of autoimmune diseases (Li et al., 2024; De et al., 2024). The formation of immune complexes initiates Fc receptor-mediated activation of plasmacytoid dendritic cells (pDCs) via endosomal toll-like receptors (TLRs), significantly increasing the production of type I interferon (IFN-I), predominantly IFN-α. Neutrophils exposed to IFN-I and immune complexes can release nucleic acids and associated proteins through mitochondrial extrusion, thereby amplifying IFN-I signaling and exacerbating disease symptoms characteristic of lupus-like conditions (Crow, 2014). Research by Sofía et al. revealed that the NDUFS4 subunit of CI can activate immune cells in conjunction with Toll-like receptor 7 (TLR7), contributing to this signaling pathway and resulting in vascular damage in lupus (Miñano et al., 2025) (Figure 1). Furthermore, Bae et al. demonstrated that in lupus-related conditions, particularly lupus nephritis, IFN-γ impairs the function of mitochondrial CI in macrophages, thereby intensifying inflammatory responses and worsening the outcomes of lupus nephritis (Bae et al., 2024) (Figure 1).

From a molecular standpoint, NLRP3 inflammasome activation is initially triggered by damage to mitochondrial components. Immunocyte pyroptosis and elevated NLRP3 inflammasome activity are correlated with long-term IFN-γ-induced suppression of mitochondrial CI. At the same time, oxidized mtDNA may leak into the cytoplasm due to mitochondrial stress or injury, activating the cGAS-STING pathway. This exacerbates lupus nephritis by encouraging the creation of IFN-I and a strong inflammatory response (Kim et al., 2023; Chen et al., 2023). CI serves as a protein implicated in the release of IFN by neutrophils. Concurrently, the suppression of CI activity in macrophages has been shown to exacerbate lupus nephritis. Consequently, CI emerges as a pertinent molecular target for the treatment of lupus nephritis, although its therapeutic efficacy and underlying mechanisms warrant further investigation.

### 3.2 Mitochondrial CI and diabetic kidney disease

Diabetes, an endocrine disorder characterized by multiple metabolic factors, has been previously discussed in relation to the association between mitochondrial gene mutations and diabetic peripheral neuropathy. A similar association is observed in other diabetic complications, such as diabetic kidney disease (DKD), a severe microvascular complication of diabetes that originates from diabetes-induced vascular changes (KDIGO, 2022, 2022). DKD remains the predominant cause of end-stage renal disease worldwide. Recent studies have identified several mtDNA variants linked to diabetes, including point mutations in transfer RNA (tRNA) genes such as MT-TL1, MT-TK, MT-TS2, and MT-TE, as well as alterations in MT-ND6, which encodes NADH:ubiquinone oxidoreductase chain 6 (Whittaker et al., 2007; Kishimoto et al., 1995). Insulin resistance is another critical factor influencing the progression of DKD (Peppa et al., 2010). Previous research has indicated that both mtDNA variants affecting CI and nuclear DNA mutations may synergistically contribute to the pathogenesis of DKD, suggesting a potential role of CI in diabetes-related renal complications (Ohkubo et al., 2000). Li et al. expanded on this by employing next-generation sequencing to examine the complete mitochondrial genomes of 50 individuals with maternally inherited diabetes, identifying five mtDNA variants encoding CI that are associated with DKD (Li et al., 2022). In their investigation of DKD, Mise et al. developed mouse models and protein interaction models, revealing that the interaction between STOML2, a podocyte mitochondrial cristae-forming protein, and NDUFS4, a ferredoxin-like protein, enhances podocyte mitochondrial cristae formation, thereby mitigating diabetic nephropathy. This relationship was further corroborated through *in vitro* cell experiments (Mise et al., 2024) (Figure 1).

From the perspectives of molecular mechanisms and pathology, cristae-forming proteins such as STOML2 play a crucial role in cristae remodeling and respiratory supercomplex assembly. When NDUFS4 expression is reduced, its interaction with STOML2 is lost, leading to mitochondrial structural remodeling. This results in disordered cristae platforms for RSC assembly, causing diminished CI function and mitochondrial dysfunction. Consequently, this exacerbates podocyte injury, thereby triggering DKD (Friedman et al., 2015). These mechanisms indicate that CI dysfunction is crucial in DKD and determines its occurrence. Patients with DKD often progress to nonfunctional kidneys, with a significantly elevated risk of requiring renal replacement therapy and kidney transplantation. This highlights the critical need for ongoing research into the molecular mechanisms underlying DKD pathobiology to identify relevant molecular targets.

### 3.3 Mitochondrial CI and kidney fibrosis

Fibrosis constitutes the pathological consequence of end-stage chronic kidney disease (CKD), characterized by renal interstitial fibrosis and glomerulosclerosis. The progression of renal fibrosis is regarded as the principal mechanism underlying the progressive decline in renal function associated with CKD (El Nahas, 2005; Eddy, 2000). Inflammatory activation, oxidative stress, and other factors are among the pathogenic contributors to renal fibrosis (Xie et al., 2009; Madesh and Hajnóczky, 2001). Despite extensive research efforts over the past decades aimed at developing enhanced therapeutic strategies for CKD, treatment outcomes have remained suboptimal, largely due to an incomplete understanding of the fundamental pathological mechanisms (Impellizzeri et al., 2014). There is an urgent need to elucidate the relevant molecular mechanisms to advance the diagnosis and treatment of renal fibrosis. Mitochondrial dysfunction disrupts the OXPHOS process, leading to ATP depletion, the release of pro-apoptotic factors, and excessive production of ROS (Figure 1). This dysfunction may result in cellular damage through oxidative damage to DNA and proteins, subsequently triggering inflammatory and fibrotic responses via the apoptotic cascade (Michel et al., 2012) (Figure 1). Mitochondrial dysfunction, particularly involving CI, is a precursor to renal fibrosis (Su et al., 2013). In their investigation of CI and obstructive urinary tract diseases, Sun and colleagues utilized mouse models, immunohistochemistry, and quantitative reverse transcription PCR (qRT-PCR) techniques. Their results demonstrate that rotenone-induced inhibition of CI dysfunction reduces oxidative stress and inflammatory responses. Moreover, the suppression of mitochondrial CI significantly attenuated fibrotic responses, as evidenced by the decreased expression of fibronectin, plasminogen activator inhibitor-1, collagen I, collagen III, and α-smooth muscle actin (α-SMA), along with a notable reduction in transforming growth factor-beta 1 (TGF-β1) levels (Sun et al., 2014a). Additionally, Shen et al. identified that Demethylzeylasteral, a CI inhibitor, can ameliorate renal interstitial fibrosis by diminishing mitochondrial-mediated oxidative stress in the kidneys (Shen P. et al., 2024).

ROS activation induced by CI dysfunction triggers the activation of transcription factors NF-κB and activator protein-1 (AP-1). AP-1 mediates the activation of the TGF-β1 promoter in mesangial cells, while ROS-induced increases in the inflammatory cytokine TNF-α accelerate this activation process. TGF-β1 induces extracellular matrix remodeling in the mesangium and triggers tubular epithelial-to-mesenchymal transition, leading to tubulointerstitial fibrosis; NF-κB enhances laminin secretion in mesangial cells (Sullivan et al., 2009; Lee et al., 2003). From the perspective of relevant mechanisms, CI plays a pivotal role in the process of renal fibrosis. Inhibiting its function can mitigate inflammatory responses and the production of ROS, which is a critical pathway in the development of fibrosis induced by renal injury. Therefore, Further research and exploration into CI-related drugs that inhibit dysfunction is urgently needed.

### 3.4 Mitochondrial CI and asthenozoospermia

Asthenospermia (AS) is a significant factor contributing to male infertility, primarily characterized by reduced sperm motility. The pathogenesis of AS is multifactorial, with inflammatory processes being a notably reversible factor affecting male reproductive function (Fathy et al., 2014; Curi et al., 2003). Inflammatory cells can compromise sperm integrity by generating ROS and pro-inflammatory cytokines, thereby diminishing sperm quality and metabolic function, ultimately resulting in male infertility. Mitochondria are regarded as a major source of oxidative stress, which may play a crucial role in human sperm defects (Armstrong et al., 1999). Under normal physiological conditions, sperm produce moderate levels of ROS, which are essential for maintaining sperm function and viability (Aitken and Drevet, 2020). ROS are critically involved in essential processes such as sperm maturation, the acrosome reaction, and sperm-egg fusion (Ochsendorf, 1999). However, excessive ROS can have deleterious effects on these sperm functions by inducing nuclear DNA strand breaks. Mitochondrial dysfunction, particularly due to CI dysfunction, can lead to elevated ROS levels, resulting in decreased sperm motility (Koppers et al., 2008). Studies suggest that mutations in CI can impair its function, further contributing to reduced sperm motility (Al Smadi et al., 2021). DJ-1, encoded by the PARK7 gene, is a 189-amino acid protein that is part of the Thi/PfPI superfamily of molecular chaperones (Inden et al., 2006). In Chinese patients with AS, DJ-1 expression is reduced in ejaculated spermatozoa. Under conditions of oxidative stress, DJ-1 relocates to the mitochondria within sperm cells, where it maintains mitochondrial integrity and protects spermatozoa from oxidative damage (Sun et al., 2014b). Wang et al. were the first to identify decreased expression of NDUFS3 in sperm from AS patients and in the testes of AS rat models. This observation suggests that reduced NDUFS3 levels in both testes and sperm may contribute to impaired sperm motility. The downregulation of NDUFS3 is likely to compromise mitochondrial CI function in testicular spermatogenic cells, leading to decreased CI activity and impaired sperm motility in AS patients (Figure 1). Experimental data indicate a strong positive correlation between the expression of DJ-1 and NDUFS3, suggesting that NDUFS3 plays a role in assisting DJ-1 in preserving mitochondrial integrity. In the context of AS, reduced levels of DJ-1, along with weakened interactions between DJ-1 and NDUFS3, may disrupt CI-associated protective mechanisms, ultimately leading to impaired sperm motility (Wang et al., 2018) (Figure 1).

Although its function in sperm mitochondria is well known, the precise method by which mitochondrial CI contributes to AS is yet unknown. DJ-1 expression is lowered and mitochondrial CI dysfunction results from reduced NDUFS3 expression. Reduced sperm motility is the result of nuclear DNA strand breakage caused by the ROS buildup that follows CI malfunction. However, more research is needed to determine the exact mechanisms driving this process. These findings highlight the potential of targeting CI as a molecular strategy for the diagnosis and treatment of AS.

## 4 Mitochondrial CI and neoplastic of urinary system diseases

Mitochondrial CI, as the primary electron acceptor in the ETC through NADH oxidation, is essential for establishing the transmembrane proton gradient across the inner mitochondrial membrane, which is crucial for ATP synthesis (Brand and Nicholls, 2011). In addition to its traditional bioenergetic function, emerging evidence indicates that CI also performs non-energetic roles that support cancer cell proliferation, growth, and metastatic potential by providing electron acceptors and regenerating oxidized cofactors (Lehuédé et al., 2016; Sullivan et al., 2015; Birsoy et al., 2015; LeBleu et al., 2014). Studies have shown that thyroid eosinophilic tumors represent a distinct category of common proliferative lesions, characterized by cells with pronounced mitochondrial hyperplasia. Gasparre et al. conducted a complete sequencing of the mtDNA in thyroid eosinophilic tumor cytopathology and identified that all disruptive mutations were located within the CI subunit gene. Furthermore, Evangelisti et al. discovered a correlation between TP53 mutations and disruptive alterations in CI genes in thyroid eosinophilic tumor samples by screening for mutations in oncogenes and tumor suppressor genes (Evangelisti et al., 2015; Gasparre et al., 2007). Additionally, research has demonstrated that the majority of mutations in breast cancer genes are predominantly located in the mitochondrial D-loop region (Tan et al., 2002). Li et al. identified that mutations in NDUFB9, an auxiliary subunit of mitochondrial CI, lead to defects in CI, thereby facilitating the proliferation of breast cancer cells (Li et al., 2015). Bastin et al. demonstrated that reduced mitochondrial CI activity in colorectal cancer cells results in elevated levels of mitochondrial ROS and disrupts NAD^+^ synthesis, which in turn limits the activity of the NAD^+^-dependent deacetylase SIRT3. Since SIRT3 activity is crucial for activating SOD2 through deacetylation, this reduction leads to decreased SOD2 enzymatic activity, thereby sustaining mtROS levels in colorectal cancer cells. These elevated mtROS may trigger the activation of FAK, which plays a role in the migration of colorectal cancer cells (Bastin et al., 2023). Furthermore, studies suggest that interactions between lysosomes and mitochondria may contribute to the progression of head and neck cancers, with mitochondrial CI showing high expression levels (Allegra et al., 2006). The S100 calcium-binding protein A4, a key protein in promoting metastasis, has been shown to upregulate the mitochondrial CI subunit Fe-S protein 2. This upregulation of mitochondrial CI enhances tumor cell invasion and metastasis in non-small cell lung cancer (Liu et al., 2019).

The involvement of mitochondrial CI in tumor cell migration, invasion, and metastasis remains a contentious topic. In certain cancer types, CI activity is elevated, whereas in others, it is reduced. This variability may be attributed to cancer-type-specific differences, but it is more likely due to the inhibitory levels of CI, which ultimately dictate its pro-tumorigenic and anti-tumorigenic effects (Tan et al., 2014; Jose and Rossignol, 2013; Benard et al., 2007). The incidence of urinary system tumors associated with mitochondrial CI has garnered increasing research interest.

### 4.1 Mitochondrial CI and renal cell carcinoma

Renal cell carcinoma (RCC) is among the most prevalent malignancies of the urinary system and is frequently encountered in clinical practice (Cancer Genome Atlas Research Network, 2013). According to the World Health Organization (WHO) classification, RCC is primarily categorized into clear cell RCC (ccRCC), papillary RCC (PRCC), and chromophobe RCC (ChRCC), accounting for approximately 65%–70%, 15%–20%, and 5%–7% of all cases, respectively (Humphrey et al., 2016). Additionally, there are other classifications, such as collecting duct and renal medullary carcinoma based on tumor anatomical location; RCC associated with acquired cystic diseases linked to renal diseases; and familial predisposition to hereditary angiomyolipomatosis (Inamura, 2017). We will primarily examine the three previously identified types of RCC. Recent research has elucidated a genetic foundation for RCC, wherein gene mutations or silencing events can promote cancer cell growth, proliferation, and invasion. For example, mutations in the TP53 and PTEN genes are prevalent, occurring in 32% and 9% of ccRCC cases, respectively (Davis et al., 2014). In the context of RCC, specific genetic alterations in genes related to mitochondrial CI may influence disease outcomes (Larman et al., 2012). In a cohort study of 61 consecutive patients with localized RCC, 34 individuals (55.7%) exhibited at least one mutation in the mitochondrial D-loop. The presence and quantity of D-loop mutations were associated with increased tumor size (>32 mm) and higher nuclear grade (≥ISUP grade 3). Importantly, the simultaneous presence of D-loop mutations and CI dehydrogenase subunit 1 mutations improved the predictive accuracy for cancer-specific mortality within the cohort, raising the concordance index (C-index) from 0.757 to 0.810 (Kim et al., 2019). This suggests that mutations in CI genes play a role in the pathogenesis of RCC.

**FIGURE 2:**
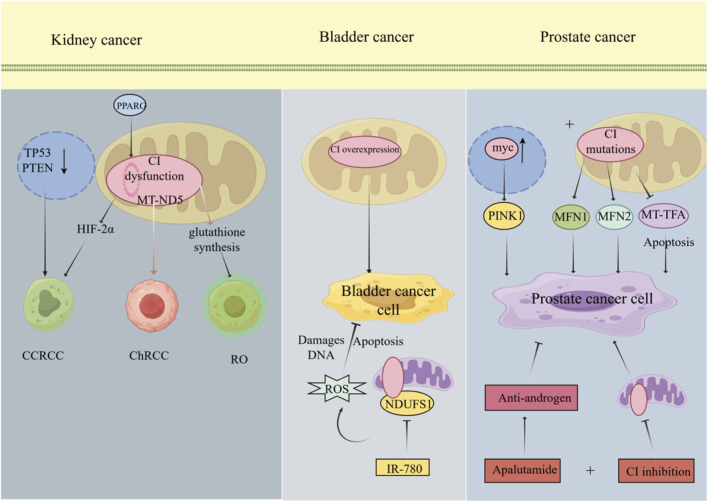
The Role of Mitochondrial CI in Tumours of the Urinary System. In renal cell carcinoma (RCC), the majority of mutations in the tumour suppressor genes TP53 and PTEN are associated with the development of clear cell RCC (ccRCC), Concurrently, reduced expression of the transcription factor PPARG decreases CI gene transcription, further activating HIF-2α to promote the progression of clear cell ccRCC; Mutations in the MT-ND5 gene can reduce CI activity, leading to chromophobe RCC; Gene mutations of CI can reduce CI activity, leading to increased glutathione synthesis and mitochondrial accumulation, thereby triggering renal oncocytoma. In bladder cancer, the chemotherapeutic agent IR-780 inhibits the activity of the NDUFS1 protein, a subunit of the CI, leading to reactive oxygen species accumulation and inducing DNA damage and apoptosis in bladder cancer cells. In prostate cancer, CI gene mutations frequently occur in tumours exhibiting high expression of the myc protein, accompanied by elevated expression of proteins such as MFN1, MFN2, and PINK1, alongside reduced MT-TFA expression. Dual therapy combining the anti-androgen drug apalutamide with a CI inhibitor effectively suppresses tumour cell progression.

In ccRCC, approximately 90% of tumors exhibit a bilateral loss of function in the gene encoding the tumor suppressor von Hippel-Lindau (Iliopoulos et al., 1996). Zhang et al. conducted an analysis using publicly accessible databases of ccRCC to examine CI expression and its associated transcription factors in both metastatic and primary ccRCC. Their findings indicated that diminished mitochondrial CI expression is associated with tumor metastasis and immune responses in ccRCC, with PPARG potentially serving as a transcriptional activator for CI genes in this context (Zhang et al., 2022) (Figure 2). Investigations involving somatic genomic sequencing of ChRCC, including mitochondrial DNA and whole-genome sequencing, have revealed reduced CI activity and mutations in the MT-ND5 gene (Davis et al., 2014) (Figure 2). Renal oncocytoma (RO) is a benign tumor of the kidney characterized by dense mitochondrial accumulation (Zambrano et al., 1999). Simonnet and colleagues examined the activity and protein content of CI in RO cells, demonstrating that the reduced levels and activity of CI protein in RO may contribute to the mitochondrial accumulation characteristic of this relatively benign tumor (Simonnet et al., 2003) (Figure 2). ChRCC and RO are closely related yet rare renal neoplasms. Although mutations in genes encoding CI significantly contribute to OXPHOS dysfunction in renal eosinophilic tumors, such genetic alterations are relatively uncommon in ChRCC. Proteomic and metabolomic analyses conducted by Xiao et al. have demonstrated that a reduction in mtDNA content, particularly the decrease in DNA encoding CI rather than CI-specific mutations, is the primary factor contributing to impaired OXPHOS in ChRCC (Xiao et al., 2020).

In ccRCC, the tumor suppressor pVHL participates in pathways associated with ccRCC development. pVHL targets the α subunit of hypoxia-inducible factor (HIF) for proteasomal degradation. Concurrently, when mitochondrial CI dysfunction occurs, HIF-2α promotes the growth, metastasis, and proliferation of ccRCC cells (Schödel et al., 2016). In ChRCC, there is currently a lack of specific mechanisms explaining how CI dysfunction contributes to its development. This stems primarily from two reasons: Firstly, ChRCC is an extremely rare cancer type that has not been extensively studied. Secondly, most clinically relevant treatments follow the standards established for ccRCC. In RO, reduced levels and activity of mitochondrial CI proteins cause mitochondrial dysfunction. This subsequently triggers significant upregulation of transcripts involved in glutathione synthesis—including the rate-limiting enzyme GCLC—enabling metabolic defense against oxidative stress and ultimately promoting RO cell growth (Lu, 2009). Collectively, these findings underscore the involvement of CI in RCC, although the precise molecular mechanisms remain to be fully elucidated. Consequently, CI emerges as a promising molecular target for both diagnostic and therapeutic strategies in RCC.

### 4.2 Mitochondrial CI and bladder cancer

Bladder cancer is among the most prevalent malignancies globally and represents the most common tumor of the urinary system. The clinical management of bladder cancer necessitates multiple therapeutic interventions, leading to significant treatment costs. Non-muscle-invasive bladder cancer (NMIBC) constitutes approximately 75% of newly diagnosed bladder cancer cases. Although the 5-year overall survival rate exceeds 80%, the disease is marked by a high recurrence rate, with up to 78% of patients experiencing relapse within 5 years (Teoh et al., 2022; Richters et al., 2020). Consequently, the identification of novel therapeutic targets for bladder cancer is of considerable importance. In this context, Shen et al. explored the potential of the chemotherapy drug IR-780 iodide, a near-infrared targeted fluorophore, for the treatment of bladder cancer. Utilizing Western blot analysis, they demonstrated that IR-780 inhibits the activity of the CI NDUFS1 protein, thereby reducing OXPHOS in bladder cancer cells. This increases the anticancer impact of IR-780 by causing excessive ROS production, which damages DNA in the relevant cancer cells and initiates apoptosis (Shen et al., 2023) (Figure 2). Consequently, CI is overexpressed in bladder cancer cells. In the context of targeted cancer cell therapy for bladder cancer, mitochondrial metabolism presents a promising avenue for tumor-specific treatment strategies (Shi et al., 2019). This is particularly relevant for drug-resistant bladder cancer cells, which frequently exhibit an increased dependence on mitochondrial function. Previous studies have demonstrated metabolic reprogramming in bladder cancer, highlighting the critical role of mitochondrial dysfunction in tumor initiation, disease progression, and the emergence of therapeutic resistance (Oresta et al., 2021; Woolbright et al., 2018; Sahu et al., 2017). Moreover, numerous chemotherapeutic agents induce apoptosis in bladder cancer cells through mitochondrial pathways (Xiao et al., 2022). Current tumor-targeted therapies have limited practical efficacy, especially in the treatment of bladder cancer. Thus, it remains to be elucidated whether CI dysfunction contributes to bladder cancer, and further investigation is required to understand the underlying mechanisms.

### 4.3 Mitochondrial CI and prostate cancer

Prostate cancer (PCa) is a major contributor to cancer-related mortality among men and is one of the most common malignancies affecting the male urinary system (Cazares et al., 2010). The etiology of PCa is multifactorial, with significant associations identified with genetic predispositions, advancing age, and obesity (Barlow and Shen, 2013). Conventional prostate-specific antigen (PSA) screening for PCa, however, has limitations, including the potential for detecting low-risk cases, leading to overdiagnosis and overtreatment (Draisma et al., 2009). Although prostate biopsy remains the definitive method for diagnosing PCa, it is not without risks and may occasionally result in missed diagnoses. The primary treatment modalities for PCa include radical prostatectomy and radiation therapy, both of which are considered curative. Nonetheless, recurrence and mortality post-treatment remain concerns for some patients (Schröder et al., 2012). There is an urgent need for the advancement of biomarkers to enhance the detection and management of PCa (Cooperberg, 2012). Notably, frequent mutations in CI have been observed in both human and Hi-myc PCa, leading to disruptions in the expression of associated complex proteins. Furthermore, depletion of mtDNA in PCa cells has been shown to compromise mitochondrial integrity, increase the expression of MFN1, MFN2, and PINK1, and decrease MT-TFA levels (Philley et al., 2016) (Figure 2). The precise processes by which MFN1 and MFN2 accelerate the development of cancer are still unknown. On the other hand, PINK1 may promote FOXO and PI 3 K/AKT signaling pathways to promote the growth of cancer (O'Flanagan and O'Neill, 2014). Reduced MT-TFA expression exerts an anti-apoptotic effect (Guo et al., 2011). In the context of therapeutic interventions, PCa cells often develop resistance to anticancer agents, presenting a significant challenge for effective disease management. While initial androgen deprivation therapy can inhibit the progression of advanced PCa, the development of resistance mechanisms allows tumor cells to survive, underscoring the necessity for alternative treatment strategies (Ward and Thompson, 2012). Baumgartner et al. reported diminished OXPHOS activity in PCa cells resistant to combined therapy with apalutamide—an anti-androgen drug—and CI inhibition, which consequently limited the proliferation, growth, and migration of these cells (Baumgartner et al., 2023) (Figure 2). Rotenone, a potent CI inhibitor, is derived from the roots of the cubeb tree and has been extensively utilized as an insecticide and piscicide. Deguelin, another compound extracted from the same source, shares a structural similarity with rotenone (Caboni et al., 2004). *In vitro* studies by Naguib et al. demonstrated that deguelin can inhibit CI, thereby reducing the growth, proliferation, and migration of PCa cells. Subsequent *in vivo* experiments in mice further indicated that deguelin can be employed to combat advanced PCa (Naguib et al., 2018). The present findings imply that alterations in mitochondrial CI may play a role in the progression of PCa. Numerous studies have demonstrated that CI enhances OXPHOS in PCa, thereby facilitating its progression. The application of specific inhibitors to target CI within the respiratory chain has shown potential in modulating PCa development (Schöpf et al., 2020; Lee et al., 2020). However, the exact molecular mechanisms through which CI influences PCa are not yet fully understood, highlighting the potential of CI as both a diagnostic and therapeutic target.

Although existing data on the prevalence of mitochondrial CI-related neoplasms within the urinary system are sparse, these initial observations suggest that CI may hold significant implications for tumor development and prognosis. Therefore, comprehensive, large-scale epidemiological studies are necessary to accurately evaluate the prevalence of these genetic alterations and their effects on clinical outcomes.

## 5 Diagnostic of mitochondrial CI in urinary system diseases

### 5.1 Molecular and biochemical biomarkers

Biomarkers indicative of mitochondrial CI dysfunction may enhance the diagnostic process for urinary system tumors. In RO, comprehensive analyses integrating exome, transcriptome, and metabolome data have identified potential biomarkers. These include frequent loss-of-function mutations in mtDNA affecting CI-encoded subunits, coupled with an upregulation of glutathione metabolism. This is evidenced by increased levels of oxidized and reduced glutathione, γ-glutamyl-cysteine, and cysteinyl-glycine, which collectively may constitute a distinct metabolic signature (Gopal et al., 2018). In the context of PCa, mutations in mitochondria-encoded respiratory complex I (RCI) and disruptions in mitochondrial integrity may also serve as biomarkers. Frequent RCI mutations observed in both human and Hi-myc PCa are associated with altered expression of the corresponding complex proteins (Philley et al., 2016). Additionally, the presence of RCI-mtDNA, MFN2, and IMMT proteins in circulating exosomes of PCa patients further underscores their potential as biomarkers.

Serum creatinine and estimated glomerular filtration rate (eGFR) are currently used in clinical practice to evaluate kidney function; however, these indicators do not indicate impairment specific to kidney function and are not sensitive for early mitochondrial CI dysfunction (Hong et al., 2018). Although it correlates with mitochondrial oxidative stress, cystatin C, a more accurate GFR biomarker that is independent of muscle mass, is unable to differentiate kidney function failure from other nephropathies (Srour et al., 2022). However, some studies suggest that there may be a potential association between the changes of biomarkers and complex I related to kidney function. On the one hand, after ischemia injury, PTECs release more neutrophil gelatinase-associated lipocalin, which is made worse by ATP depletion and oxidative stress brought on by suppression of mitochondrial CI (Chen et al., 2018). Elevated urine NGAL levels precede histological damage in CI-deficient Ndufs4^−/−^ mice, suggesting vulnerability to bioenergetic dysfunction (Shrestha et al., 2012). On the other hand, Complex I disassembly in kidney injury molecule-1 (KIM-1) shedding and loss of mitochondrial membrane potential are correlated with phosphatidylserine receptor overexpression in damaged PTECs (Li et al., 2021). Importantly, KIM-1 ectodomain shedding requires ATP-dependent metalloproteinase activity, thereby functionally linking its release to the cellular energy state governed by mitochondrial Complex I. Research has identified urinary ATP synthase subunit β (ATPSβ) as a potential indicator of renal mitochondrial dysfunction in cases of acute kidney injury (AKI). In murine models subjected to ischemia/reperfusion-induced AKI, immunoblot analyses demonstrated increased levels of both full-length and cleaved forms of urinary ATPSβ, which correlated with mitochondrial impairment. Similarly, elevated urinary ATPSβ levels were detected in patients who developed AKI following cardiac surgery, reinforcing its potential as a non-invasive biomarker (Whitaker et al., 2015).

Consequently, mitochondrial dysfunction plays a critical role in various urological diseases, including both neoplastic and non-neoplastic conditions. Further investigation into the mechanisms of mitochondrial dysfunction in urological diseases could solidify its role as a biomarker for diagnosing these conditions.

### 5.2 Imaging and functional assessment techniques

Imaging modalities provide critical insights into mitochondrial CI function in non-neoplastic urinary diseases. In a murine model exhibiting partial CI deficiency due to Ndufs6 gene trap insertion, renal dysfunction was evident, indicating that imaging techniques might be capable of detecting early signs of mitochondrial impairment. Both Ndufs6^gt/+ and Ndufs6^gt/gt mice demonstrated typical renal pathology features, such as albuminuria, increased urinary excretion of kidney injury molecule-1, and renal fibrosis (Vaidya et al., 2006; Forbes et al., 2013). These imaging techniques hold promise for visualizing these changes and monitoring disease progression associated with CI deficiency. Multiphoton microscopy has been effectively utilized to evaluate mitochondrial structure and function in rodent models of acute kidney injury. In anesthetized animals, mitochondrial dynamics and functional status can be assessed using multiphoton excitation of both endogenous and exogenous fluorophores. For instance, ischemia resulted in a significant elevation in mitochondrial nicotinamide adenine dinucleotide levels and a rapid loss of mitochondrial membrane potential in proximal tubules (Hall et al., 2009; Hall et al., 2013). These methodologies can be adapted to explore non-neoplastic urinary diseases linked to CI dysfunction, offering real-time insights into mitochondrial changes. Despite the current scarcity of techniques specifically targeting mitochondrial CI, existing imaging strategies present promising opportunities for future research and clinical applications.

### 5.3 Genetic and omics diagnostic strategies

Molecular diagnostics play a crucial role in identifying mitochondrial CI-related diseases within the urinary system. Patient-derived fibroblast cell lines with CI defects can be transduced with either wild-type or mutant NDUFS1 cDNA, followed by comprehensive functional and proteomic analyses (Danhauser et al., 2011). This approach enables the assessment of the pathogenicity of rare variants, which is vital for accurate molecular diagnosis. Furthermore, exome sequencing has been utilized to diagnose CI deficiencies. In a study involving ten unrelated individuals with CI deficiency, exome sequencing, in conjunction with sequential bioinformatic filtering, was employed to identify candidate variants (Haack et al., 2012). Subsequent cellular rescue experiments validated the pathogenicity of novel alleles. These methodologies facilitate the swift identification of pathogenic variants in both well-established and newly discovered genes associated with CI, thereby improving the molecular diagnosis of urinary system diseases related to mitochondrial CI dysfunction.

## 6 Therapeutic strategies targeting mitochondrial CI in urinary system diseases

### 6.1 Pharmacological inhibitors

Pharmacological strategies targeting mitochondrial CI in urinary tumors have shown potential. In RCC, the inhibition of prenylation has been observed to enhance the efficacy of chemotherapy. The HMG-CoA reductase inhibitor, pitavastatin, obstructs the mevalonate pathway, thereby inhibiting protein prenylation. Additionally, pitavastatin disrupts mitochondrial respiration by suppressing complexes I and II, diminishes glycolytic flux, and consequently induces significant energy depletion and oxidative stress. Importantly, the combination of pitavastatin with paclitaxel has demonstrated superior efficacy compared to either agent alone in both *in vitro* cell cultures and *in vivo* RCC xenograft models (Whyte et al., 1997; Huang et al., 2017). In the context of bladder cancer, hyperbaric oxygen has been shown to enhance the chemotherapeutic efficacy of the mitochondria-targeting compound IR-780. This compound preferentially accumulates in bladder cancer cells and induces apoptosis by targeting the mitochondrial CI subunit NDUFS1. Concurrent treatment with hyperbaric oxygen significantly amplified the anti-tumor effects of IR-780 *in vitro* by facilitating cellular uptake and inducing excessive mitochondrial ROS generation. This combination also effectively suppressed tumor growth and recurrence in animal models without evident toxicity (Shen et al., 2023). Furthermore, fibrate lipid-lowering agents, such as fenofibrate, have been explored for their potential antitumor effects in bladder cancer. Fenofibrate was found to inhibit mitochondrial CI activity, leading to reduced ATP production, ROS accumulation, and disruption of the mitochondrial membrane. Additionally, it activated the AMPK/mTOR signaling pathway and downregulated CD276 expression, thereby enhancing T cell-mediated antitumor immunity (Li et al., 2025). Collectively, these findings suggest that pharmacological targeting of mitochondrial CI may constitute a promising therapeutic strategy for the treatment of urinary tumors.

### 6.2 Protective agents and function-enhancing strategies

Non-pharmacological strategies targeting the modulation of mitochondrial CI function in non-neoplastic diseases are receiving increasing scholarly attention. The reversible glutathionylation of CI has been demonstrated to enhance mitochondrial superoxide production (Taylor et al., 2003). Understanding this mechanism could aid in the development of non-pharmacological approaches to modulate mitochondrial CI function. For example, interventions that regulate the redox balance of the mitochondrial glutathione pool may modulate CI activity and alleviate oxidative stress associated with non-neoplastic diseases. In a murine model of nonalcoholic fatty liver disease (ob/ob mice), the expression levels of mitochondrial CI subunits were found to be reduced; however, treatment with uric acid, anti-TNFα antibodies, or a manganese superoxide dismutase mimetic successfully restored normal CI function (García-Ruiz et al., 2010). While these findings were derived from liver disease models, similar strategies may be applicable to non-neoplastic diseases of the urinary system. Such interventions have the potential to restore mitochondrial CI function and improve disease outcomes in the urinary tract. N-acetylcysteine (NAC) has been shown to mitigate rotenone-induced CI dysfunction in THP-1 cells. Rotenone exposure led to increased mitochondrial superoxide production, elevated levels of cell-free mitochondrial DNA, and upregulation of the NDUFS7 subunit (Kwok et al., 2023). Pre-treatment with NAC reduced the rotenone-induced elevation of cell-free mitochondrial DNA and NDUFS7 protein levels, suggesting that NAC may protect mitochondrial CI from rotenone-induced dysfunction. Genetic factors also play a role in CI integrity; for example, APOL1 renal-risk variants are known to impair mitochondrial function. In doxycycline-inducible HEK293 Tet-on cells stably expressing APOL1 G0, G1, or G2, the expression of the G1 or G2 variants, driven by doxycycline, significantly decreased maximal respiration, reduced spare respiratory capacity, and compromised mitochondrial membrane potential (Ma et al., 2017). The findings suggest that APOL1 renal-risk variants may play a role in the pathogenesis of mitochondrial CI-related kidney diseases. Furthermore, chronic low-level expression of IFN-γ has been demonstrated to impair CI activity in renal macrophages, potentially acting as an early mechanistic driver of lupus nephritis. In ARE-Del mice, a model characterized by sustained low-level IFN-γ expression and lupus nephritis-like phenotypes, age- and tissue-specific gene expression analyses revealed significant suppression of mitochondrial CI components and enzymatic activity, particularly within the kidneys (Bae et al., 2024). Understanding these genetic and environmental risk factors is crucial for developing preventive strategies and implementing early interventions for urinary system diseases associated with mitochondrial CI dysfunction.

### 6.3 Emerging therapies

Emerging therapeutic strategies targeting mitochondrial CI in urinary system diseases are garnering significant scholarly interest. Approaches centered on mitochondrial function, including the use of naturally derived compounds and innovative techniques such as fusion proteins, are currently under investigation for the treatment of CKD. Given that mitochondrial dysfunction is a pivotal factor in the pathogenesis of CKD, these interventions aim to rectify abnormalities such as increased oxidative stress, impaired mitochondrial biogenesis, excessive mitochondrial fission, and dysregulated mitophagy (Sun et al., 2025). In a murine model exhibiting mitochondrial CI deficiency, the neuroprotective CI inhibitor CP2 demonstrated partial reversal of molecular alterations akin to those found in Alzheimer’s disease (Gao et al., 2025). While these findings are derived from research on Alzheimer’s disease, the concept of utilizing targeted inhibitors to rectify mitochondrial CI dysfunction may be applicable to diseases of the urinary system. Analogous inhibitors could be developed to restore CI function in renal or urinary tract cells that are affected. Furthermore, the TAT-mediated protein transduction system emerges as a promising therapeutic platform for addressing CI deficiency and other mitochondrial pathologies. Proteins fused with the transduction domain of the HIV-1 transactivator of transcription (TAT) have the capability to penetrate cells and restore the function of deficient cargo proteins in cells derived from patients (Lin and Kao, 2015). This methodology could be employed to deliver functional mitochondrial CI subunits or related proteins to cells within the urinary system, presenting a potential therapeutic strategy for conditions resulting from CI deficiency.

### 6.4 Mitochondrial CI and kidney protection

Oxidative stress is closely associated with mitochondrial CI dysfunction in diseases of the urinary system. In rat models infused with aldosterone, the administration of rotenone, a specific inhibitor of CI, was found to attenuate renal injury by reducing oxidative stress, mitochondrial dysfunction, and inflammasome activation. Rats treated with aldosterone displayed glomerular segmental sclerosis, foot process effacement, and proteinuria, which were accompanied by increased markers of oxidative stress, diminished ATP levels, and a reduced mtDNA copy number. The administration of rotenone significantly ameliorated these pathological alterations, suggesting that oxidative stress mediated by mitochondrial CI plays a role in aldosterone-induced renal damage (Distelmaier et al., 2009). In models of gentamicin-induced nephrotoxicity, sitagliptin was shown to mitigate renal mitochondrial dysfunction and apoptosis, effects that were associated with a reduction in oxidative stress (Ding et al., 2015). The administration of gentamicin led to significant increases in blood urea nitrogen, serum creatinine, and urinary protein levels, alongside reduced activities of antioxidant enzymes and heightened oxidative stress markers. Sitagliptin treatment reversed these changes, suggesting its potential for renal protection by alleviating oxidative stress linked to mitochondrial CI dysfunction (Abuelezz et al., 2016). In models of maleate-induced renal injury, curcumin provided nephroprotection by reducing mitochondrial fission and autophagy, which were associated with decreased oxidative stress. Maleate exposure increased superoxide anion production and facilitated the formation of protein adducts, whereas curcumin treatment ameliorated these oxidative changes (Molina-Jijón et al., 2016). These findings collectively highlight the pivotal role of mitochondrial CI in regulating oxidative stress in urinary system diseases and suggest that targeting CI could be a promising therapeutic strategy for reducing oxidative stress-induced renal injury.

### 6.5 Challenges and outlook

Although the therapeutic targeting of mitochondrial CI in illnesses of the urinary system has shown significant preclinical promise, there are significant obstacles in the way of its translation into clinical practice. This chapter outlines three crucial issues that must be resolved in order to fully utilize mitochondrial CI modulation techniques: target specificity, delivery obstacles, and treatment resistance.

Since CI is expressed in every nucleated cell, specific therapeutic targeting is inherently difficult. The dose-limiting toxicities seen in early-phase trials of IACS-010759, such as nausea, vomiting, and cardiac arrhythmias, show that the danger of on-target, off-tumor toxicity is especially severe for systemic CI inhibitors (Yap et al., 2015). The critical involvement of CI in high-energy-demand tissues, especially heart muscle, neurons, and renal tubules, is reflected in these negative impacts. Due to its tissue-specific expression patterns and varied incorporation, the NDUFA4 subunit has historically been categorized as a CI component (Carroll et al., 2003). The treatment window may be expanded by specifically targeting NDUFA4-containing Cl complexes that are abundant in particular tumor subtypes. Additionally, options for subtype-selective inhibition are presented by newly discovered CI assembly intermediates and supercomplex topologies exclusive to cancer cells.

Drug penetration across the outer and inner membranes of the mitochondria, as well as the plasma membrane, is necessary for effective CI targeting. The hydrophobic inner membrane restricts the distribution of aqueous drugs, while the very negative mitochondrial membrane potential prevents the accumulation of anionic substances (Murphy, 2008). The inefficient mitochondrial partitioning of current CI inhibitors, such as rotenoids, biguanides, and IACS-010759, necessitates high systemic dosages that worsen off-target effects.

Through metabolic reprogramming, even cancers that were originally receptive develop adaptive resistance. Long-term CI inhibition favors (Viale et al., 2014): PGC-1α-mediated upregulation of mtDNA replication and respiratory chain components; increased glutaminolysis, fatty acid oxidation, or macropinocytosis to maintain ATP and biosynthetic precursors (Garcia-Bermudez et al., 2022); mitophagy suppression: accumulation of malfunctioning mitochondria with decreased drug sensitivity (Palikaras et al., 2015). All of these contribute to the increase in drug resistance.

## 7 Discussion

### 7.1 Debates on the role of mitochondrial CI in urinary system tumour progression

The role of CI in the progression of urinary system tumors continues to be a subject of debate. In PCa, there is evidence indicating that alterations in CI, coupled with increased mitochondrial fusion, are associated with tumor progression; however, the exact mechanisms by which CI mutations contribute to oncogenesis remain inadequately elucidated. Studies analyzing mtDNA in both human and murine models of PCa have demonstrated that recurrent respiratory CI (RCI) mutations disrupt the expression of the associated complex proteins (Jerónimo et al., 2001). Furthermore, the depletion of mtDNA in PCa cells has been shown to compromise mitochondrial integrity, highlighting the significant impact of mtDNA-related alterations on CI function and their potential role in prostate cancer development. Although recurrent RCI mutations in both human and Hi-myc PCa disrupt the expression of the corresponding complex proteins, it remains unclear whether these mutations act as primary drivers of tumor progression or occur as secondary effects (Philley et al., 2016). In RO, while loss-of-function mtDNA mutations in genes encoding CI are common, the subsequent events and their precise contributions to tumor development are not fully understood. The interplay between CI loss, chromosome 1 loss, and cyclin remains to be clarified (Gopal et al., 2018; Gasparre et al., 2008). A growing body of research indicates that CI has a “double-edged sword” role in cancer biology, either promoting or suppressing tumorigenesis based on microenvironmental cues, metabolic state, and genetic background. This dichotomy can be explained by two non-exclusive theories.

The Metabolic Flexibility Threshold Hypothesis. According to this theory, CI inhibition changes from cytoprotective to cytotoxic when tumor cells reach a “threshold” of metabolic flexibility (Liberti and Locasale, 2016). Compensatory mechanisms, especially glycolysis and glutaminolysis, adequately maintain ATP and biosynthetic precursors at mild CI dysfunction, enabling adaptive survival. However, this threshold is crossed by significant CI impairment, which results in oxidative stress, cell death, and a catastrophic energy crisis (Yang et al., 2014). Recurrent RCI mutations in prostate cancer paradoxically increase mitochondrial fusion and tumor invasiveness while disrupting holoenzyme assembly, indicating that partial rather than complete CI loss gives a selection advantage. On the other hand, in PTEN-deficient mice, pharmacological CI inhibitors such rotenone and deguelin, cause total enzymatic blockage, overwhelming compensatory capability and causing synthetic lethality (Molina et al., 2018). According to recent research, primary ccRCC lesions show suppressed tricarboxylic acid (TCA) cycle labeling, which is typified by significantly lower glucose-derived contributions to TCA cycle intermediates and decreased ETC activity, specifically compromised CI function. Metastatic lesions, on the other hand, show increased TCA cycle labeling. Remarkably, OXPHOS is upregulated in metastatic locations, and CI mostly promotes metastasis through NAD^+^ regeneration rather than direct ATP synthesis. This further shows that metastasis is blocked below a threshold of CI activity, which must be reached for metastasis to occur (Bezwada et al., 2024). The metabolic flexibility threshold concept is further supported by this discovery.

The Tumour Microenvironment Dependency Hypothesis. An alternate approach highlights that microenvironmental restrictions, including oxygen tension, food availability, and immune infiltration composition, determine the oncogenic versus tumor-suppressive effects of CI (Li et al., 2019). Aspartate synthesis and nucleotide biosynthesis are supported by HIF-independent CI activity in normoxic, glucose-replete circumstances, which are typical of early-stage ccRCC and fuel proliferation (Birsoy et al., 2015). However, CI-derived reactive oxygen species (ROS) stimulate the production of vascular endothelial growth factor (VEGF) and stabilize HIF-1α in the hypoxic, acidic environment of advanced tumors, thus encouraging angiogenesis and metastasis (Chandel et al., 1998). Spatial heterogeneity is revealed by recent single-cell metabolomic analyses: CI-low cells occupy hypoxic cores and exhibit phenotypes resistant to therapy, while CI-high populations predominate perfused, normoxic habitats and display stemness markers (Hensley et al., 2016). This spatial partitioning implies that intratumoral zonation needs to be taken into consideration in CI modulation techniques.

These frameworks function at several biological scales rather than being mutually exclusive. While microenvironment reliance encompasses paracrine and systemic interactions, the metabolic flexibility threshold mainly deals with cell-autonomous responses to CI disruption. In the end, combining multi-omics profiling with spatially defined functional assays will be necessary to address the CI dilemma in urological oncology.

### 7.2 Comparative and integrative perspective: the CI functionin in urinary pathology

Mechanistic Divergence: Energy Crisis or Biosynthetic Hijacking. CI dysfunction starts a disastrous energy cascade in benign urine diseases. Proton pumping efficiency is compromised by genetic mutations in NDUFS subunits or mtDNA-encoded genes (MT-ND1/2/3/4/5/6), which collapse the ATP production and mitochondrial membrane potential (Distelmaier et al., 2009). Pyruvate dehydrogenase and tricarboxylic acid cycle flow are inhibited by the ensuing NADH accumulation, resulting in ineffective glycolysis that is unable to satisfy bioenergetic demands (Liberti and Locasale, 2016). Superoxide anions are produced concurrently with electron leakage at defective CI, overriding antioxidant defenses and causing lipid peroxidation, DNA damage, and inflammasome activation. Cristae architecture is disrupted by NDUFS4 loss in diabetic kidney disease, intensifying this vicious cycle (Mise et al., 2024). Therefore, maintaining residual CI activity, improving mitophagy quality control, and replenishing glutathione pools constitute the restorative therapeutic need (Shen P. et al., 2024). On the other hand, cancer cells use CI activity to meet their anabolic needs. Urinary carcinomas still have a strong OXPHOS capacity, according to contemporary metabolomics, despite the Warburg effect’s historical emphasis on glycolysis (Faubert et al., 2017). Asparagine synthetase and dihydroorotate dehydrogenase processes depend on CI-derived NAD^+^, which connects respiratory chain function to nucleotide and protein production (Sullivan et al., 2015). Through PGC-1α coactivation, HIF-2α activation paradoxically raises CI expression in clear cell RCC, resulting in a “pseudo-hypoxic” metabolic state that facilitates metastatic colonization (Zhang et al., 2022). Pharmacological inhibition is the therapeutic window in this case: acute CI blockage causes ferroptosis, replication stress, and the collapse of aspartate pools in PTEN-deficient prostate cancer cells (Molina et al., 2018).

The CI Function Continuum Hypothesis. We suggest that these seeming inconsistencies resolve along a continuum of CI functions. Severe CI impairment (>70% activity loss) results in energetic catastrophe and cell death at the loss-of-function extreme; this is compatible with the growth of indolent oncocytomas but incompatible with aggressive malignancy. The biosynthetic pathways required for fast proliferation are supported by intact CI at the maintained-function extreme. The intermediate zone is where the therapeutic sweet spot is found: partial CI inhibition (30%–70% activity reduction) that surpasses the ability of cancer cells to compensate while protecting healthy tissues. A number of clinical observations are explained by this continuum. First, because total CI loss is incompatible with malignant fitness, germline CI mutations result in Leigh syndrome or oncocytomas, which are never aggressive carcinomas (Marin et al., 2013). Second, pharmacological CI inhibitors show cancer-selective toxicity: normal cells activate adaptive autophagy and survive, while cancer cells are susceptible to acute energetic stress due to their fast ATP turnover and low metabolic reserve (He et al., 2015). Third, baseline CI expression is correlated with the effectiveness of CI-targeted therapies: MT-ND5-mutant ChRCC exhibits intrinsic resistance (Xiao et al., 2020).

This integrated approach raises a number of problems. (1) What epigenetic processes allow cancer cells to continue expressing CI in the face of hypoxic stress? (2) Is it possible to create “metabolic switches” that alter CI activity according to tissue environment using synthetic biology techniques? (3) How is the CI continuum altered by the tumour microenvironment, particularly cancer-associated fibroblast metabolism? Multi-omic profiling of organoids produced from patients, spatial metabolomics, and dynamic metabolic imaging will be necessary to address issues.

### 7.3 Challenges in targeting mitochondrial CI for non-neoplastic urinary diseases

The understanding of the pathophysiology of systemic opportunism is incomplete. MedicineTargeting mitochondrial CI for non-neoplastic urinary diseases faces several challenges. In the case of mitochondrial CI deficiency-related non-neoplastic diseases, the pathophysiological mechanism is still poorly understood. For example, in children with isolated, nuclear-encoded CI deficiency, although a homogeneous clinical picture has been observed, the exact connection between the genetic mutations, cellular pathology, and the resulting clinical signs is not fully clear (Distelmaier et al., 2009). This lack of understanding makes it difficult to develop targeted and effective therapies.

Off-target Effects and the Specific Opportunities of Targeted Delivery Technologies. Another challenge is the specificity of targeting. Many potential therapeutic agents that target mitochondrial CI may also affect other cellular processes, leading to off-target effects. For example, some inhibitors of mitochondrial CI may also impact other components of the electron transport chain or cellular metabolism, which could cause adverse effects. Systemic toxicity is a danger associated with the lack of tissue specificity of several pharmacological CI modulators. Despite having strong CI inhibition, rotenone produces parkinsonian neurotoxicity by passing through the blood-brain barrier (Cann et al., 2009). This restriction has spurred advancements in medication delivery that targets mitochondria. When paired with renal clearance optimization, TPP^+^ (triphenylphosphonium)-conjugated drugs accumulate 100–500 times in mitochondria based on membrane potential, allowing kidney-selective administration (Kelso et al., 2001). Developing agents that specifically target mitochondrial CI in non-neoplastic urinary diseases without significant off-target effects is a major challenge.

In addition, the heterogeneity of non-neoplastic urinary diseases related to mitochondrial CI dysfunction poses a challenge. The development of universal treatment options may be complicated by patient variability in genetic mutations and underlying mechanisms that contribute to mitochondrial CI failure. Personalized therapeutic approaches, designed according to each patient’s unique genetic profile and pathophysiological context, will likely be essential; however, their implementation requires further investigation and advances in technology. Crucially, the resolution of these challenges in non-neoplastic diseases will inform cancer therapy development. Kidney-specific CI delivery technologies, for instance, may be repurposed for tumour-targeted inhibition, while precision stratification methods developed for benign diseases could predict cancer patient responses to CI blockade.

### 7.4 Future research directions for mitochondrial CI in urinary system health and disease

Future research on mitochondrial CI in the context of urinary system function and pathology should focus on several critical areas. Firstly, comprehensive investigations are needed to elucidate the specific role of mitochondrial CI in maintaining normal urinary system physiology. This entails examining its interactions with other cellular components and metabolic pathways within renal and other urinary system cells. A thorough understanding of its normal function will provide a solid foundation for comprehending how dysfunction may lead to disease. Secondly, extensive epidemiological studies are essential to accurately determine the prevalence of mitochondrial CI-associated urinary system diseases, including both neoplastic and non-neoplastic conditions. Such studies will facilitate the development of early detection and prevention strategies. Additionally, the identification of specific biomarkers for mitochondrial CI dysfunction in urinary system diseases will enhance diagnostic precision and enable earlier intervention. Thirdly, research should aim to develop more targeted and effective therapeutic strategies. This may entail the development of pharmacological agents that selectively target mitochondrial CI while minimizing off-target effects, alongside the investigation of non-pharmacological strategies such as gene therapy and cell-based interventions. A comprehensive understanding of the molecular mechanisms underlying mitochondrial CI assembly and function will be essential for the rational design of these therapeutic approaches. Furthermore, research should also prioritize the examination of the long-term implications of targeting mitochondrial CI, including potential adverse effects and the emergence of resistance, to ensure the safety and efficacy of prospective treatments.

### 7.5 Translational medicine perspectives: from mechanisms to clinical applications

At this crucial juncture in the understanding of mitochondrial CI biology in urinary diseases, mechanistic discoveries must be translated into therapeutically useful instruments. This section outlines specific approaches to using CI as a biomarker platform and combining CI-targeted modalities with conventional treatments.

Mitochondrial CI as a Biomarker for Clinical Stratification and Prognosis. The first is the liquid biopsy method. Non-invasive options are provided by circulating biomarkers. A proxy indicator of mitochondrial turnover, urinary ATP synthase subunit β increases dramatically in acute renal damage and is correlated with CI-dependent energy stress (Whitaker et al., 2015). Lupus nephritis flare-ups are predicted by cell-free mtDNA (cf-mtDNA) in plasma, which is measured by droplet digital PCR of MT-ND1 or MT-CO1 regions and represents tissue-specific CI failure (Caielli et al., 2016). Single-molecule sequencing of cf-mtDNA can now reveal heteroplasmic CI mutations with >0.1% allele frequency thanks to advanced platforms, potentially detecting subclinical disease prior to organ injury (Guo et al., 2009). The second is Metabolic Imaging. Static biomarkers are not the same as functional imaging. Reduced flux in prostate cancer xenografts predicts response to CI inhibitors; hyperpolarized [1–^13^C]pyruvate MRI shows real-time pyruvate-to-lactate flux, an indirect readout of CI activity (Brindle et al., 2011). By mapping the mitochondrial redox state with subcellular resolution, NADH fluorescence lifetime microscopy (FLIM) can differentiate between necrotic core and viable tumor areas (Walsh et al., 2013). Adaptive dosing and dynamic therapy monitoring may be made possible by these technologies, which are presently in phase I/II trials.

Synergistic Combinations: CI-Targeted Therapies with Conventional Treatments. (1) CI Inhibition and Immune Checkpoint Blockade. Combining CI dysfunction with immune checkpoint inhibitors (ICIs) is justified by its immunomodulatory effects. CI inhibition enhances antigen presentation and T cell priming by increasing tumor cell mtDNA release, which activates cGAS-STING signaling and type I interferon production (White et al., 2014). In preclinical models of bladder and prostate cancer, rotenone works in concert with anti-PD-1 antibodies to provide full responses that are not possible with either drug alone (Vardhana et al., 2020). But timing is crucial: sequential dose (CI inhibitor → ICI after metabolic priming) maximizes the therapeutic index, while concurrent delivery runs the risk of severe toxicity (Champiat et al., 2018). (2) Radiotherapy Sensitization. By stopping radiation-induced mitochondrial biogenesis and repopulation, CI inhibition makes hypoxic tumor cells more susceptible to radiation. Compared to historical controls, hypofractionated radiation therapy plus ME-344 (a CI inhibitor) produces greater complete response rates in muscle-invasive bladder cancer (Shen et al., 2023). Persistent DNA damage signaling and poor homologous recombination repair as a result of NAD^+^ depletion are part of the mechanism (Park et al., 2016). (3) CI Modulation and Targeted Therapy. By downregulating PGC-1α, HIF-2α inhibitors (belzutifan) indirectly reduce CI in renal cell carcinoma; when combined with direct CI inhibitors, they cause synthetic lethality in VHL-deficient models (Chen et al., 2016). Androgen deprivation therapy (ADT) for prostate cancer increases OXPHOS as a compensatory survival mechanism; in castration-resistant disease, adding deguelin or metformin (an indirect CI inhibitor) avoids ADT resistance and extends progression-free survival (Schöpf et al., 2020).

## 8 Conclusion

Mitochondrial CI occupies a central role in the physiology and pathology of the urinary system. In various non-neoplastic conditions, such as lupus nephritis, diabetic kidney disease, renal fibrosis, and asthenozoospermia, CI dysfunction leads to a collapse in energy supply, induces oxidative stress, and exacerbates inflammation, thereby serving as a common pathway to tissue damage (Table 1). In contrast, within renal, bladder, and prostate cancers, CI activity is exploited to promote metastasis and resistance to chemotherapy. However, targeted inhibition of CI can induce lethal ROS bursts in malignant cells (Table 1). In urology, mitochondrial CI research is just getting started. A greater understanding of this paradox—that the same molecular machinery may both create and heal disease—leads to a paradigm that is therapeutically accessible, biomarker-guided, and environment-specific. Sustained investment in mitochondrial biology, interdisciplinary cooperation between nephrologists, urologists, oncologists, and metabolic scientists, and audacious clinical trial designs that take into account the complexity of the diseases we aim to eradicate are all necessary to realize this ambition. Patients who are waiting for these developments—those with inevitable metastatic prostate cancer, recurrent bladder cancer, or progressive diabetic nephropathy—deserve our wholehearted dedication to converting the information contained in this review into real, long-lasting clinical improvements.

**TABLE 1:** Role of Mitochondrial CI in urinary system diseases.

Disease type	Mitochondrial CI expression	Key molecules involved	Expression of key molecules	Evidence type	Role	References
Lupus nephritis	High Low	NDUFS4, IFN-I IFN-γ	High High	Mouse model Mouse model	High expression of NDUFS4 promotes the secretion of INF-I by inflammatory cells, further stimulating autoantibody production and leading to lupus nephritis IFN-γ expression disrupts mitochondrial complex I activity in renal macrophages, thereby triggering lupus nephritis	Crow, 2014; Miñano et al. (2025) Bae et al. (2024)
Diabetic kidney disease	Low	NDUFS4 STOML2	Low	Mouse model	Low expression of Ndufs4 in podocytes disrupts the integrity of the podocyte foot process and promotes the progression of diabetic kidney disease	Mise et al. (2024)
Kidney fibrosis	Low	ROS	High	Mouse model	CI dysfunction promotes renal inflammatory responses and apoptosis, ultimately leading to renal injury and subsequent fibrosis	Michel et al. (2012), Su et al. (2013)
Asthenozoospermia	Low	DJ-1, NDUFS3	Low	Mouse model and patient samples	Reduced expression of NDUFS3 leads to mitochondrial CI dysfunction in spermatocytes, while concurrently low DJ-1 expression further impairs mitochondrial integrity, thereby causing diminished sperm quality	Sun et al. (2014b), Wang et al. (2018)
Clear cell RCC	Low	PPARG	Low	Public ccRCC Database	Low PPARG expression and reduced mitochondrial CI expression in renal clear cell carcinoma promote metastasis of renal clear cell carcinoma	Zhang et al. (2022)
Chromophobe RCC	High	MT-ND5	Low	Cell line study	Mutations in the MT-ND5 gene lead to decreased mitochondrial CI activity but increased expression, ultimately contributing to the growth, proliferation, and metastasis of ChRCC cells	Davis et al. (2014)
Renal oncocytoma	Low	MT-ND1, MT-ND5, MT-ND4, MT-ND3	Low	patient samples	Mutations in key molecules lead to mitochondrial CI loss, further promoting glutathione metabolism in renal eosinophilic cell tumors, thereby inducing tumor cell growth and proliferation	Gopal et al. (2018)
Bladder cancer	High	NDUFS1	High	Mouse model	Increased expression of the mitochondrial CI NDUFS1 protein elevates OXPHOS levels in bladder cancer cells. This leads to reduced reactive oxygen species production, thereby enhancing the growth, proliferation, and metastasis of bladder cancer cells	Shen et al. (2023)
Prostate cancer	Low	MFN1, MFN2, PINK1 MT-TFA	High Low	Mouse model and patient samples	Reduced expression of mitochondrial CI compromises mitochondrial integrity in Pca cells, upregulates MFN1, MFN2, and PINK1 expression, and simultaneously lowers MT-TFA levels, thereby promoting the growth, proliferation, and metastasis of Pca cells	Philley et al. (2016)
